# Tethered Cord Syndrome in Pediatric and Adult Populations: A Retrospective Analysis of Outcomes and Associated Spinal Dysraphisms

**DOI:** 10.7759/cureus.102084

**Published:** 2026-01-22

**Authors:** Ahtesham Khizar, Faiqa I Khan, Haseeb Mehmood Qadri, Hassaan Zahid, Ammad Abid, Omer B Adnan, Maryem Tanweer, Haysum Khan, Muhammad Fawad Ul Hassan, Sundas Irshad, Asif Bashir

**Affiliations:** 1 Neurological Surgery, Punjab Institute of Neurosciences, Lahore, PAK; 2 General Surgery, Lahore General Hospital, Lahore, PAK; 3 Medicine, Mayo Hospital/King Edward Medical University, Lahore, PAK; 4 Neurological Surgery, Punjab institute of Neurosciences, Lahore, PAK; 5 Neurological Surgery, Mayo Hospital/King Edward Medical University, Lahore, PAK; 6 Medicine, Punjab Institute of Neurosciences, Lahore, PAK; 7 Neurological Surgery, Shifa Tameer-E-Millat University Shifa College of Medicine, Islamabad, PAK; 8 Neurological Surgery, National Hospital and Medical Centre, Lahore, PAK

**Keywords:** bladder dysfunction, low and middle country (lmic), neurological outcome, pakistan, postoperative complications, spinal dysraphism, surgical detethering, tethered cord syndrome

## Abstract

Background and objective

Tethered cord syndrome (TCS) is an amalgam of neurologic, urologic, orthopedic, and dermatologic dysfunctions with concurrent spinal dysraphism and deformities. Data from Pakistan regarding the surgical management of TCS remain limited. This study aimed to evaluate the clinical and functional outcomes of surgical detethering in patients with TCS and spinal dysraphism.

Materials and methods

This retrospective study was conducted at the Punjab Institute of Neurosciences (PINS), Lahore, Pakistan. We analyzed the outcomes of 21 patients (12 pediatric and nine adults) with TCS who were operated on between January 2020 and June 2025. Patient records were reviewed using the institution’s Picture Archiving and Communication System (PACS), operative notes, and medical charts. Results of the treatment were summarized and analyzed using descriptive statistical analysis.

Results

Among the cohort, 12 patients were pediatric and nine were adults. Pediatric patients had an average age of 4.95 ± 5.03 years and showed a female predominance (66.66%, n = 8). Adults had a mean age of 23.44 ± 8.84 years and also demonstrated a female predominance (66.66%, n = 6). Lower limb weakness was the most frequent presenting symptom, occurring in 50% (n = 6) of children and in a higher proportion of adults at 66.66% (n = 6). Lipomyelomeningocele was the most frequently observed form of spinal dysraphism in children, accounting for 33.33% (n = 4), whereas thickened filum terminale was most common in adults, seen in 33.33% (n = 3). The conus level was most commonly located at L1 in pediatric patients, observed in 50.00% (n = 6), while in adults it was most frequently at L3, occurring in 55.55% (n = 5). Detethering of the spinal cord was the predominant surgical intervention in both pediatric and adult groups, performed in 91.66% (n = 11) of children and 100% (n = 9) of adults. Postoperative assessment showed neurological improvement in 75% (n = 9) of pediatric patients and 55.55% (n = 5) of adults, with no major complications reported in 91.66% (n = 11) of children and 66.66% (n = 6) of adults.

Conclusions

Surgical untethering in patients with TCS leads to improvement in neurological function with an acceptable safety profile, highlighting the importance of early intervention.

## Introduction

Tethered cord syndrome (TCS) is a clinical disorder that develops as a result of spinal cord compression caused by abnormal stretching [1]. Common symptoms include neurological deficits in the lower limbs, spastic gait, neurogenic bladder, bowel dysfunction, and either scoliosis or foot deformities [2]. TCS predominantly occurs in childhood or adolescence and shows a male predilection [3]. The incidence is reported to be 0.25 per 1,000 births [4]. Potential causes include a thickened filum, lipomas, meningoceles, adhesions, and spinal cord malformations [1].

Neurological assessments and imaging are the primary methods of diagnosing TCS [3]. MRI remains the gold standard for confirming TCS [5]. Management is largely surgical, with untethering procedures required to improve neurological function, alleviate symptoms, and prevent further deterioration [2]. However, the optimal timing for surgical intervention remains a matter of debate [2]. Potential surgical complications include infection, cerebrospinal fluid (CSF) leak, and injury to nerve roots or the spinal cord [4].

This case series involves patients with TCS treated at the largest public-sector neuroscience center in Pakistan. To the best of our knowledge, no previous case series or published data on TCS from Pakistan have appeared in a PubMed-indexed journal. This study aimed to evaluate outcomes of surgical management of TCS to contribute to the regional literature. These findings may guide the diagnosis and treatment of the condition in comparable healthcare settings.

## Materials and methods

This study was designed as a retrospective, descriptive analysis. It was conducted at the Department of Neurosurgery, Unit-I, Punjab Institute of Neurosciences (PINS), Lahore, Pakistan, primarily involving two lead surgeons. The study included all pediatric and adult patients diagnosed with TCS and coexisting spinal dysraphisms who presented between January 1, 2020, and June 30, 2025. Patient data were retrieved from institutional records without any direct patient contact. A non-probability, consecutive sampling technique was used to include all eligible cases presenting during the specified period.

Inclusion and exclusion criteria

All patients with radiological and/or intraoperative evidence of symptomatic primary spinal dysraphism, such as lipomyelomeningocele (LMMC), dermal sinus tract, split cord malformation, or thickened filum terminale, were included. Only those who received neurosurgical evaluation and treatment at PINS and had complete clinical, radiological, and operative records available in the institutional database were considered. Patients with isolated TCS without associated spinal dysraphism, those who had prior spinal dysraphism surgery at another institution, and cases with incomplete or missing clinical or imaging records were excluded. Cases in which the diagnosis was uncertain or not confirmed by radiology or intraoperative findings were also excluded.

Operational definitions

The authors considered a patient to have motor improvement if muscle power increased by at least one grade according to the Medical Research Council (MRC) Scale for Muscle Strength. For sensory examination, an improvement in the sensory component was noted if the patient’s ability to process sensory information was better after surgery compared to baseline. Bladder control improvement was assessed by a reduction in incontinence episodes and the patient’s ability to control voiding or defecation. Pain relief was reported as ‘improved’ or ‘pain relieved’ if there was at least a 2-point decrease in the pain score on a 1-10 scale for adults or the Wong-Baker FACES scale for non-toddler children, in admitted patients, along with a reduction in pain medication use during follow-up. Overall status improvement was assigned to patients who achieved favorable outcomes after surgery, such as returning to work or performing daily life activities efficiently.

Data were collected retrospectively using a structured data collection tool via Google Forms, designed specifically for this research. Patient records were reviewed using the institution’s Picture Archiving and Communication System (PACS), operative notes, and medical charts stored in the hospital’s internal digital database. All data were analyzed using SPSS Statistics version 29.0 (IBM Corp., Armonk, NY). Descriptive statistics were used to analyze the data: categorical variables were presented as frequencies and percentages, and continuous variables were reported as mean ± standard deviation (SD) or median with interquartile range (IQR), depending on data distribution. 

Ethical considerations

The study was approved by the Institutional Review Board (IRB) of the Punjab Institute of Neurosciences under the reference number 2243/IRB/PINS/Approval/2025, dated July 02, 2025.

## Results

A total of 21 patients were included in this study, of which 12 were pediatric, and nine were adult, as summarized in Tables 1-2.

**Table 1:** Summary of included pediatric patients managed surgically for tethered cord syndrome M: male; F: female; SSI: surgical site infection; LBP: low back pain *=Standard terms used for generalizability. The assessment of patients’ signs and symptoms was based upon caregiver-reported improvement in discomfort and resolution of pain-associated behaviors as reported by the parents.

Pt. #	Age (years, y)	Gender (M/F)	Associated spinal dysraphism and deformities	Postoperative complications	Functional status at 3rd month
1	0.33 y (4 mo)	F	Myelomeningocele	SSI	Sensorimotor and bladder control improvement, LBP relieved*
2	9 y	F	Diastematomyelia	Nil	Sphincter dysfunction is the same as preoperatively
3	1 y	M	Dermal sinus tract	Nil	LBP relieved*
4	0.5 y (6 mo)	F	Myelomeningocele	Nil	LBP relieved*
5	1 y	M	Lipomyelomeningocele	Nil	Sensorimotor improvement*
6	2 y	F	Myelomeningocele	Nil	Motor improvement, bladder control, sensory improvement
7	2 y	F	Lipomyelomeningocele	Nil	Motor and bladder control improvement*
8	0.5 y (6 mo)	F	Lipomyelomeningocele	Nil	Motor improvement, pain relieved*
9	12 y	F	Scoliosis with diastematomyelia	Nil	Motor improvement
10	12 y	M	Dermal sinus tract	Nil	LBP relieved
11	12 y	M	Lipomyelomeningocele	Nil	LBP relieved
12	7 y	F	Diastematomyelia	Nil	Motor improvement, bladder control

**Table 2:** Summary of included adult patients managed surgically for tethered cord syndrome. M: male; F: female; SSI: surgical site infection; CSF: cerebrospinal fluid; LBP: low back pain

Pt. #	Age (y)	Gender (M/F)	Associated spinal dysraphism and deformities	Postoperative complications	Functional status at 3rd month
1	21	F	Dermal sinus tract	SSI	Motor improvement, LBP relieved, sensory improvement
2	17	F	Diastematomyelia	SSI, CSF leak	Motor improvement, LBP relieved, sensory improvement
3	28	M	Thickened filum terminale	Nil	Motor improvement, LBP relieved
4	44	M	Meningocele	Nil	Motor improvement, bladder control
5	17	F	Lipoma	Nil	Same as before
6	17	F	Diastematomyelia	Nil	Bladder control, LBP relieved
7	20	F	Thickened filum terminale	Nil	No improvement in LBP
8	19	F	Thickened filum terminale	Nil	LBP relieved
9	28	M	Lipoma	Nil	Motor improvement

In both the pediatric and adult populations, a predominance of females was observed. Most patients presented with lower limb weakness with an approximate duration of three to four years. Demographic characteristics and clinical data are summarized in Table 3, which compares pediatric and adult patients.

**Table 3:** Patient demographics, presenting symptoms, duration, and clinical findings on examination SD: standard deviation

Variable	Pediatric	Adults
Age, years, mean ± SD	4.95 ± 5.03	23.44 ± 8.84
Gender predilection, n (%)	Female, 8 (66.66%)	Female, 6 (66.66%)
Duration of symptoms on average, years	3.92	4.40
Presenting signs and symptoms, n (%)		
Lower back pain	6 (50.00%)	6 (66.66%)
Lower limb weakness	6 (50.00%)	6 (66.66%)
Bladder dysfunction	4 (33.33%)	3 (33.33%)
Cutaneous manifestation	4 (33.33%)	2 (22.22%)
Spinal deformity	1 (8.33%)	1 (11.11%)
Bowel dysfunction	Nil	1 (11.11%)
Sensory level, n (%)		
D10	Nil	1 (11.11%)
D12	2 (16.66%)	1 (11.11%)
L1	7 (58.33%)	2 (22.22%)
L2	1 (8.33%)	1 (11.11%)
L3	2 (16.66%)	4 (44.44%)

In our case series, lipomyelomeningocele was the most common finding in the pediatric population, in contrast to thick filum in adults. Conus level and other MRI findings are documented in Table 4.

**Table 4:** Types of spinal dysraphism and level of conus

Variable	Pediatrics, n (%)	Adults, n (%)
Types of spinal dysraphism		
Lipomyelomeningocele	4 (33.33%)	2 (22.22%)
Myelomeningocele	3 (25.00%)	Nil
Dermal sinus tract	2 (16.66%)	1 (11.11%)
Diastematomyelia	3 (25.00%)	2 (22.22%)
Scoliosis	1 (8.33%)	Nil
Meningocele	Nil	1 (11.11%)
Thickened filum terminale	Nil	3 (33.33%)
Syrinx or hydromyelia	Nil	Nil
Level of conus		
L1	6 (50.00%)	2 (22.22%)
L2	Nil	1 (11.11%)
L3	4 (33.33%)	5 (55.55%)
L4	2 (16.66%)	Nil
L5	Nil	1 (11.11%)

Untethering was done in almost all patients along with addressing the underlying pathology. Dural repair was done where necessary. Only one adult patient had a cerebrospinal fluid (CSF) leak postoperatively. Procedural details, along with intraoperative findings and clinical outcomes, are documented in Table 5. A descriptive comparison of preoperative and postoperative clinical features is presented in Table 6 for both pediatric and adult cohorts.

**Table 5:** Procedural and postoperative outcome details of patients with tethered cord syndrome SSI: surgical site infection; CSF: cerebrospinal fluid

Variable	Pediatrics, n (%)	Adults, n (%)
Type of procedure		
Untethering of the cord	11 (91.66%)	9 (100%)
Excision of meningocele	5 (41.66%)	1 (11.11%)
Dural repair	3 (25.00%)	1 (11.11%)
Excision of dermal sinus	1 (8.33%)	1 (11.11%)
Resection of filum	Nil	1 (11.11%)
Postoperative neurological status		
Sensori-motor improvement	7 (58.33%)	4 (44.44%)
Bladder control	4 (33.33%)	Nil
Lower back pain relief	5 (41.66%)	3 (33.33%)
Postoperative functional outcomes		
Status improved	9 (75.00%)	5 (55.55%)
Unchanged neurology (same as preoperative findings)	3 (25.00%)	4 (44.44%)
Postoperative complications		
SSI	1 (8.33%)	2 (22.22%)
CSF leak	Nil	1 (11.11%)
Motor deficit	1 (8.33%)	Nil
None	11 (91.66%)	6 (66.66%)

**Table 6:** Descriptive comparison of preoperative and postoperative clinical features in pediatric and adult cohorts

Variable	Pediatrics, n (%)		Adults, n (%)	
	Preoperative	Postoperative	Preoperative	Postoperative
Lower back pain	6 (50.00%)	5 (41.66%)	6 (66.66%)	3 (33.33%)
Lower limb weakness	6 (50.00%)	7 (58.33%)	6 (66.66%)	4 (44.44%)
Bladder dysfunction	4 (33.33%)	4 (33.33%)		3 (33.33%)

**Figure 1:**
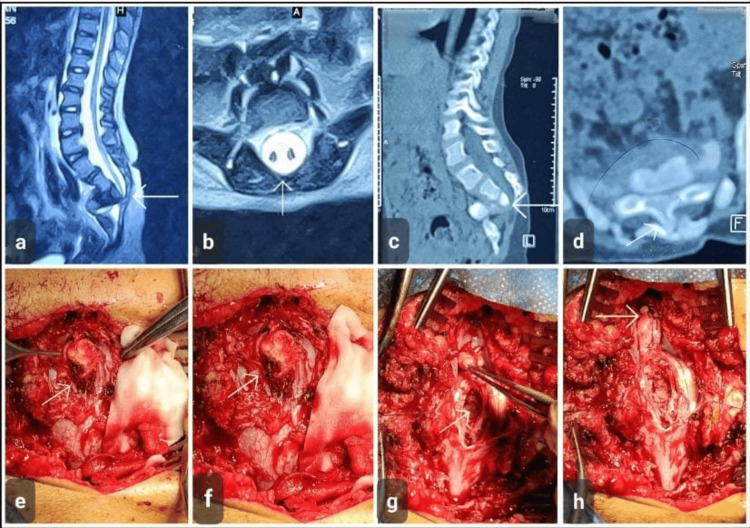
Case 1 Seven-year-old female with diastematomyelia. (a) Sagittal T2-weighted MRI of the lumbosacral spine demonstrating a split spinal cord with associated intradural abnormality (arrow). (b) Axial T2-weighted MRI showing duplication of the spinal cord within a single dural sac, consistent with diastematomyelia (arrow). (c) Sagittal CT scan revealing a bony septum extending into the spinal canal (arrow). (d) Axial CT image confirming the presence of an osseous spur causing division of the spinal canal (arrow). (e-h) Intraoperative photographs illustrating surgical exposure of the split cord malformation, identification of the bony septum (arrows), excision of the spur, subsequent decompression, division of filum terminale, and restoration of a single dural sac MRI: magnetic resonance imaging; CT: computed tomography

**Figure 2:**
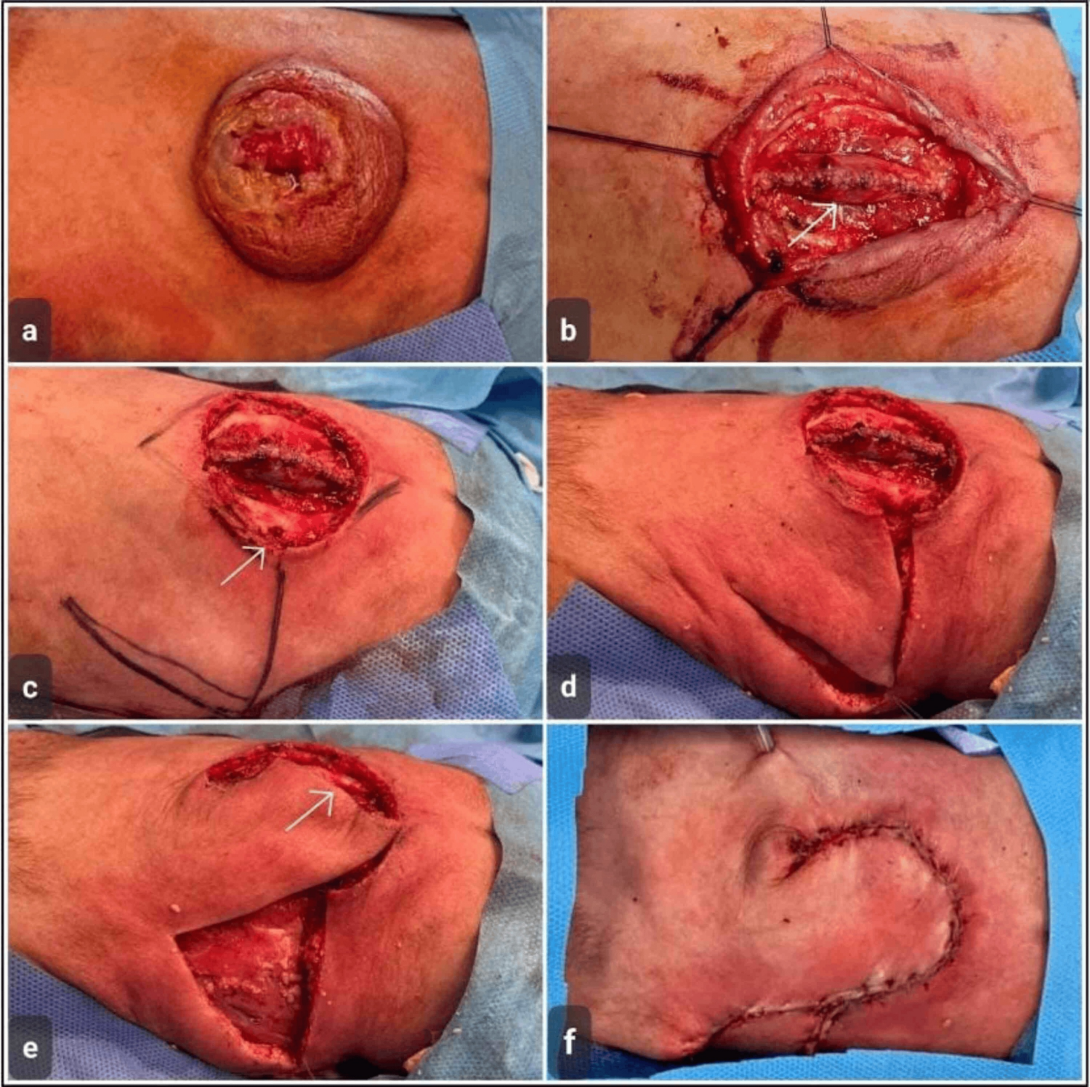
Case 2 Surgical repair of myelomeningocele using a rotational skin flap in a four-month-old female. (a) Preoperative view demonstrating a lumbosacral myelomeningocele sac with overlying skin breakdown. (b) Intraoperative exposure following incision, showing the dural repair around the neural placode, and surrounding dysplastic tissue (arrow). (c-d) Mobilization of the surrounding skin and subcutaneous tissue with delineation of the rotational flap margins (arrow). (e) Rotation of the flap to cover the central defect after neural tissue repair (arrow). (f) Final positioning of the rotational flap, achieving tension-free coverage of the myelomeningocele defect with layered wound closure

**Figure 3:**
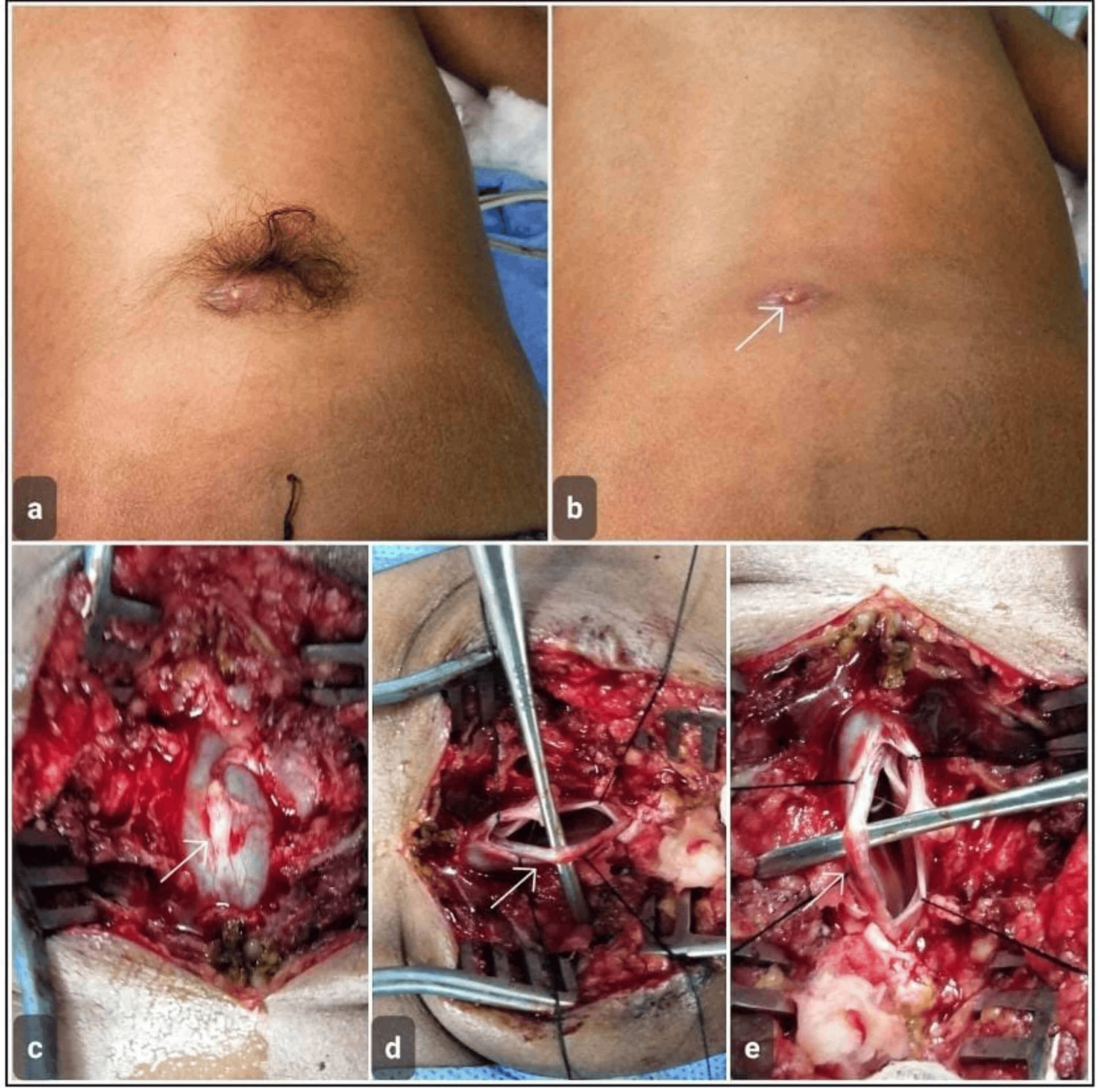
Case 3 Dermal sinus tract with intradural extension in a one-year-old male. (a) Preoperative clinical photograph showing a midline cutaneous pit with the surrounding hair tuft in the lumbosacral region. (b) Close-up view highlighting the dermal sinus opening after hair removal (arrow). (c) Intraoperative exposure demonstrating the sinus tract extending through the subcutaneous tissues toward the spinal canal (arrow). (d) Microsurgical dissection of the dermal sinus tract with identification of its intradural component (arrow). (e) Complete excision of the sinus tract with division of filum terminale (arrow)

**Figure 4:**
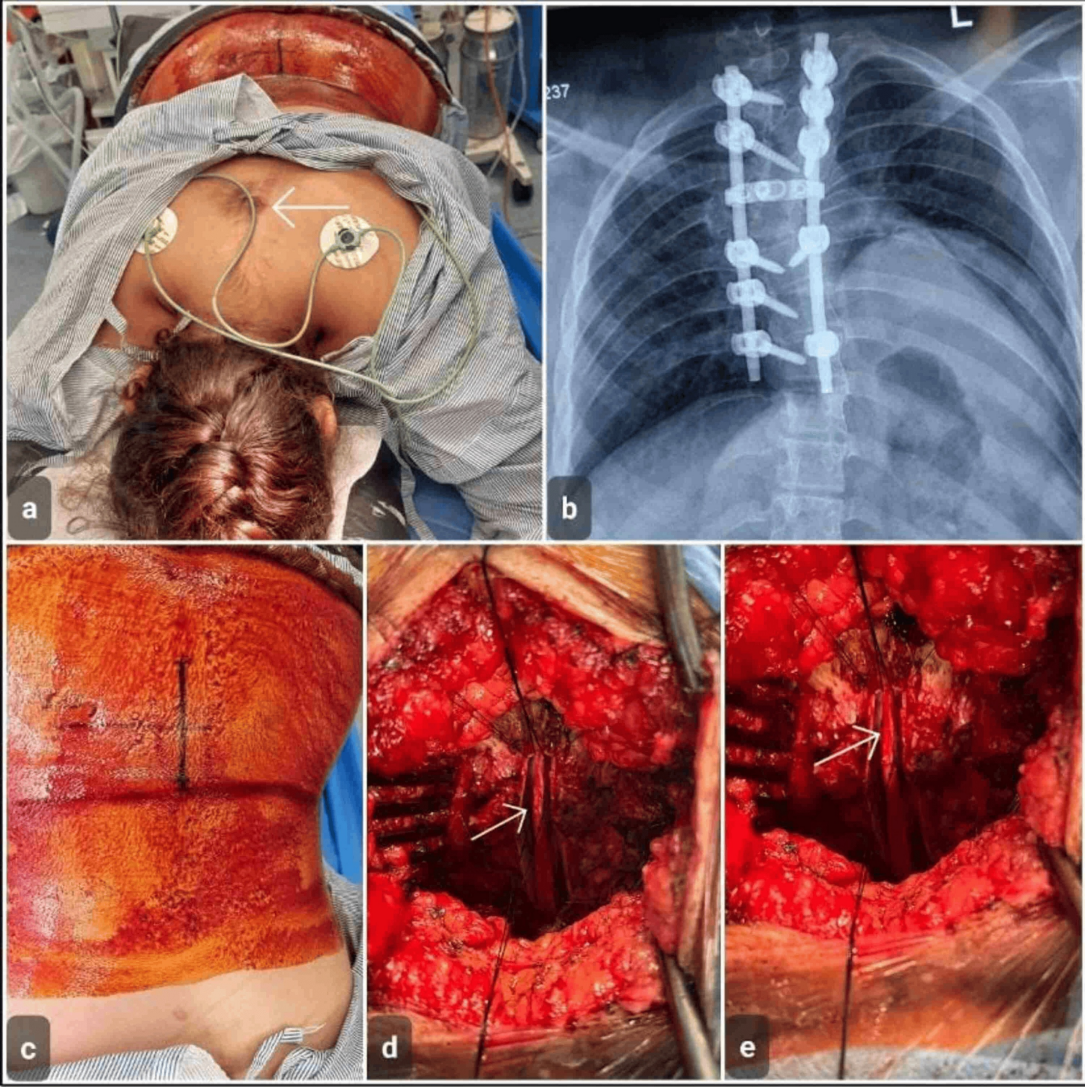
Case 4 Previously operated dorsal diastematomyelia with tethered spinal cord in a 20-year-old female. (a) Preoperative photograph showing a healed midline dorsal surgical scar from prior spinal surgery (arrow). (b) Posteroanterior radiograph of the thoracic spine demonstrating posterior spinal instrumentation from the previous procedure done in an orthopedic facility. (c) Intraoperative view after skin preparation and midline incision marking. (d) Intraoperative microscopic view demonstrating tethered neural elements (arrow). (e) Microsurgical untethering of the spinal cord (arrow)

**Figure 5:**
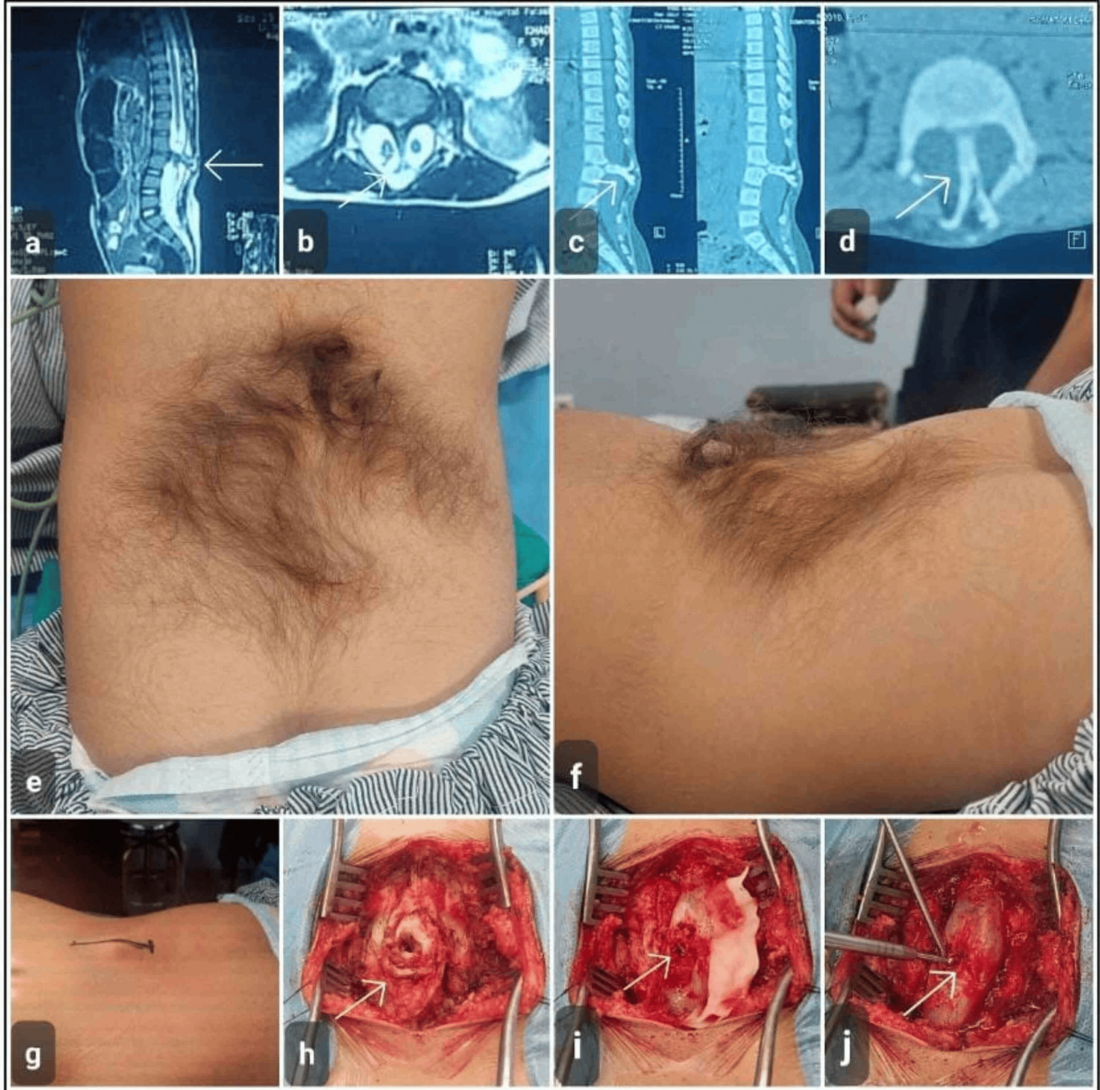
Case 5 Diastematomyelia with cutaneous stigmata and intraoperative findings in a nine-year-old girl. (a) Sagittal T2-weighted MRI demonstrating a split spinal cord with associated intradural abnormality (arrow). (b) Axial T2-weighted MRI showing duplication of the spinal cord within the spinal canal, consistent with diastematomyelia (arrow). (c) Sagittal CT scan revealing an osseous spur projecting into the spinal canal (arrow). (d) Axial CT image confirming the bony septum dividing the spinal canal (arrow). (e-f) Preoperative clinical photographs demonstrating a midline lumbosacral cutaneous stigma with a tuft of hair, suggestive of underlying spinal dysraphism. (g) Surface marking of the planned midline surgical incision. (h-i) Intraoperative exposure showing the bony septum (arrow). (j) Drilling of the bony septum (arrow), and then the dural sac is opened, microsurgical untethering of the cord is performed and repair done in a single dural sac MRI: magnetic resonance imaging; CT: computed tomography

We present five illustrative cases in Figures 1-5.

## Discussion

Tethering occurs when the filum (distal end of the cord) anchors caudally. A constellation of symptoms appears due to the stretch on the cord and roots; the resulting phenomenon is the so-called tethered cord syndrome. Tethering usually occurs due to an underlying occult spinal dysraphism (OSD), such as a thick filum, split cord, lipomyelomeningocele, etc. [6]. Acquired TCS can be attributed to a spinal tumor or trauma. However, congenital TCS arises due to an aberration in primary neurulation. Several contributors have been proposed, including genetic factors (60-70%), maternal obesity and diabetes, and folate deficiency [7]. Its incidence and associated mortality have declined to 8%; however, it still occurs at a rate of 18.6 per 10,000 births worldwide [7].

This retrospective study aimed to evaluate the preoperative spectrum and postoperative outcomes in patients with TCS. The study population comprised a total of 21 patients, of whom 12 (57.14%) were pediatric and nine (42.85%) were adult patients. The mean age at the time of presentation was 4.95 ± 5.03 years in the pediatric population, slightly lower than that reported in a systematic review from 2024 (6.4 ± 5.5 years) [8]. The mean age of adult patients in our study was 23.44 ± 8.84 years, younger than that reported in the existing literature (36.2 ± 15.7 years) [8]. The younger age observed in our cohort could be attributed to early recognition of motor weakness in six (50.00%) pediatric and six (66.66%) adult patients, which was first brought to attention in otherwise healthy individuals. There was a female gender predominance in both groups, pediatric (n = 8, 66.66%) and adult (n = 6, 66.66%). This gender predilection differs from the literature, perhaps due to the small sample size [1,8]. The median interval between the onset of symptoms and presentation to the hospital was 4.40 years in adults, in accordance with O’Connor et al.’s meta-analysis from 2020 (4.7 years) [1]. In comparison, the median interval in the pediatric group was 3.92 years.

The most common presenting symptoms in both groups were pain and lower limb weakness, with six cases (50.00%) in the pediatric group and six cases (66.66%) in adults. A 2024 systematic review of 3315 cases by Kun He et al. revealed bladder dysfunction (34.8%) as the most common symptom in the pediatric population, followed by cutaneous stigmata (28.6%), bowel dysfunction (13.9%), and motor deficit (12.8%) [8]. In our cohort, bladder dysfunction and cutaneous stigmata were the second most frequent findings after pain and motor weakness, each observed in four cases (33.33%). In an Egyptian study by Elmesallamy et al., back pain was the most common symptom (100%), followed by urinary abnormality (75%), lump (70%), paresthesia (67%), and limb weakness (61%) [9]. Lower levels of the conus (L5 and S1) in previous studies have been correlated with more pronounced symptoms [8,9]. Conversely, in our study, the level of the conus was L1 in six (50%) cases, which was normal for the mean age of 4.95 ± 5.03 years. As a result, the symptoms were subtle and were recognized only after gross weakness developed. Pain is a confusing and unreliable parameter in children. Notably, in our study, LMMC in four (33.33%) and myelomeningocele (MMC) in three (25.00%) were more prevalent in the pediatric population, suggesting that the conus extended beyond the boundaries of the spinal canal, thus causing the symptomatology. This was somewhat similar to Kun He et al.'s findings, where TCS secondary to LMMC or MMC was more common [8].

That said, the literature divides TCS into primary forms (thick or tight filum) and secondary forms (due to spinal dysraphism, MMC, etc.) [8]. Primary TCS is more common in adults, and the caudal level of the conus is typically at the L3 to L5 level. Literature suggests that the more distal the level of the conus, the greater the stretch on the cord, and therefore the more pronounced and earlier the symptoms. In our study, the conus level in adults was at L3 to L5 in five cases (55.55%), and primary TCS was more common in the form of a thick filum, in accordance with international literature. Pain in six patients (66.66%) and lower limb weakness in six patients (66.66%) were the most frequent presenting features, followed by bladder dysfunction in three patients (33.33%). Previous systematic reviews showed urinary abnormalities as the most frequently reported symptom, which warrants further evaluation for spinal dysraphism in adults undergoing urodynamic studies [8]. However, in a meta-analysis, O’Connor et al. reported lower back pain (81%), followed by motor deficit (63%) and bladder dysfunction (56%), as the most troubling symptoms, consistent with our findings [1].

Adults are more likely to report distressing pain and subtle changes in limb power as compared to children. In contrast, Elmesallamy et al. reported urinary abnormality in 100% and back pain in 80% of cases [9]. In a Pakistani cohort of 50 patients, leg pain and urological problems were more common [10]. However, in the study by Ali et al., LMMC and MMC were more common than a thick filum [10]. In contrast, in our study, two cases (22.22%) of LMMC and no cases of MMC were identified, which can be attributed to our small sample size of nine adult patients, limiting generalizability to the broader population. Furthermore, Habib et al. discussed the possibility of TCS as a differential diagnosis in patients presenting with constipation [11]. However, in our cohort, we did not encounter any patient with such a complaint.

Among other spinal dysraphisms, diastematomyelia, or split cord syndrome (SCS), was present in three (25.00%) pediatric and two (22.22%) adult patients. SCS can be type I, in which a bony spur divides the cord into two halves within two separate dural sleeves, and type II, in which a fibrous band divides the cord into two halves encased in the same dural sheath [12]. The SCS case series by Kobets et al. showed seven (77.77%) cases with type II and two (22.22%) with type I presentations. A bony spur was found at the lower lumbar or sacral levels (L5 to S2, L3 to L5), while a fibrous band in type II was located slightly higher (T12 to L5) [12]. Pain was the most common presenting feature in this case series as well, consistent with our findings. In our study, a dermal sinus was identified in two (16.66%) pediatric and one (11.11%) adult patient. This number was lower than that reported in a similar country cohort of 110 patients by Ahmed et al., in which nine (8.7%) had a dermal sinus [13]. Additionally, orthopedic manifestations, such as clubfoot (talipes equinovarus), talipes equinus, or cavus foot, are common in spinal dysraphism, but were not observed in this cohort, owing to the small sample size.

The mainstay of treatment is tethered cord release (TCR) to avoid permanent neurological damage [8,9,13]. Michael et al., in their critical analysis, emphasized patient-oriented decisions [14]. Stretching of the cord causes hypoxic and metabolic insults to the cells, resulting in disruption of normal function [10]. The literature reports dramatic improvement in pain [8,9,10]. In our cohort, untethering was performed in all cases, including 11 (91.66%) pediatric and nine (100%) adult patients. Excision of MMC, LMMC, and dermal sinus was performed when indicated, along with dural repair. Intraoperative electrophysiological monitoring can aid surgical planning, although it is currently not used in our center [15]. Despite this limitation, in our study, motor sensory improvement was more common in children (n = 7, 58.33%) and adults (n = 4, 44.44%), followed by pain relief in five (41.66%) pediatric and three (33.33%) adult patients.

Elmesallamy et al. reported pain improvement in 60% of children and 75% of adults, followed by bladder function improvement in 60% of children and 30% of adults [9]. Bladder control was achieved in only four (33.33%) pediatric patients and in none of the adult patients in our study. Ali et al. also reported significant pain improvement (78%) and urological improvement in 50% of patients [10]. This can be attributed to the importance of early surgery and the release of cord stretch, which alleviates pain. Improvement in motor weakness has been reported to occur more frequently in adults compared to children [8]. This, however, can be attributed to earlier recognition of weakness in adults before permanent damage occurs.

In our study, three (25.00%) pediatric patients and four (44.44%) adult patients remained at their preoperative status. A meta-analysis, with a mean patient age of 35.6 years, reported unchanged pain status in 13% and worsened pain status in 2% [1]. Moreover, urological status remained unchanged in 41% and worsened in 2% [1], while motor deficits remained unchanged in 25% and worsened in 3% [1]. This suggests that early detection and intervention can reverse developing, potentially temporary deficits [3]. Wound infection occurred in one (8.33%) pediatric and two (22.22%) adult patients, followed by one (11.11%) adult patient who developed a postoperative CSF leak. These complications were somewhat lower than those reported in the literature, likely due to meticulous surgical technique and the limited sample size. Ahmed et al. reported two (8.7%) readmissions due to CSF leak requiring surgical repair [13].

Bhimani et al. documented reoperation in 101 patients (2.7%), with CSF leaks being the most common cause, followed by superficial wound infection in 3.1% and deep wound disruption in 1.4% during a 30-day follow-up [4]. CSF leaks are less frequently encountered, but they remain the most common cause of reoperation after TCR. Long-term follow-up is necessary to monitor the development of any symptoms, as scar formation can lead to secondary tethering [8]. Iqbal et al. proposed a Karachi TCS severity score, which is based on gait, power, sensation, and bowel/urinary function, with scores ranging from 5 to 0 to indicate increasing severity [16]. In that study, improvement in scores was observed after surgery during 12 to 48 months of follow-up [16].

Limitations

Our study was limited by its small sample size and retrospective design. Considering the rarity of the disease and the data collection method, the true population may not have been fully captured. Nevertheless, our results demonstrate that the sample accurately represents the demographics, clinical features, and outcomes of the targeted pathology. However, long-term follow-up is lacking, which prevents a full understanding of the natural progression of the disease after detethering.

Recommendations

A multicenter prospective study with a larger patient sample and longer follow-up is necessary for more reliable outcome assessment. As demonstrated in our study, early diagnosis and timely treatment are crucial for improving prognosis. Even with limited resources, countries like Pakistan can achieve progress by strengthening referral systems. For example, any patient presenting with neurological symptoms, regardless of age or duration, should be referred from primary or secondary care directly to a tertiary hospital, with an MRI performed as the immediate next step. Prognosis depends not only on early surgical intervention but also on the expertise and skill of the neurosurgical team. For better outcomes, these cases should be managed by experienced teams. Implementing a standardized scoring system to monitor symptom progression or improvement would also be beneficial. Future research should compare outcomes between patients receiving prophylactic surgery for tethered cord syndrome and those operated on after symptom onset, to better evaluate the benefits and risks of early intervention.

## Conclusions

TCS includes a spectrum of disorders with neurological, urological, and orthopedic manifestations. Primary TCS can present in children and young adults. Although pain and motor weakness are evident in both age groups, bowel and bladder dysfunction are more pronounced in adults. Cutaneous stigmata facilitated early diagnosis in the pediatric group, while in adults, a thick filum gradually leads to symptom development. Tethering can occur even when the conus is within normal limits in patients with symptomatic dysraphism. Moreover, early detection and release of the tethered cord remain central to achieving a favorable outcome. Persistence or worsening of symptoms after surgical release highlights the importance of microsurgical techniques and intraoperative neuromonitoring. Furthermore, long-term follow-up is essential to monitor for acquired tethering or improvement of symptoms over time.
